# Machine learning-based predictive model for the perioperative co-occurrence of T-cell-mediated rejection and pneumonia in liver transplantation

**DOI:** 10.3389/fimmu.2025.1648993

**Published:** 2025-09-17

**Authors:** Junjie Sun, Guangyi Zhu, Qingwen Liang, Ning Wen, Haibin Li, Xuyong Sun

**Affiliations:** Institute of Transplant Medicine, The Second Affiliated Hospital of Guangxi Medical University, Guangxi Clinical Research Center for Organ Transplantation, Guangxi Key Laboratory of Organ Donation and Transplantation, Nanning, China

**Keywords:** machine learning, liver transplantation, T-cell-mediated rejection, pneumonia, perioperative period, predictive model

## Abstract

**Objective:**

Perioperative T-cell-mediated rejection (TCMR) and pneumonia occurrence significantly impair graft function and patient survival following liver transplantation (LT). This article aims to develop a machine learning (ML)-based model to predict perioperative co-occurrence of TCMR and pneumonia.

**Methods:**

Recipient-related data were retrospectively collected. Predictive Variables were identified through LASSO regression analysis. Five machine learning algorithms, including support vector machine (SVM), were employed to develop predictive models. Model performance was appraised via the receiver operating characteristic (ROC) curve, and calibration curve. SHapley Additive exPlanations (SHAP) method was employed to visualize model characteristics and individual predictions.

**Results:**

This study enrolled 717 LT recipients, including 93 patients with perioperative co-occurrence of TCMR and pneumonia. LASSO regression identified postoperative direct bilirubin, postoperative international normalized ratio, high-density lipoprotein, postoperative alanine aminotransferase, natural killer cell, tacrolimus (FK506) concentration, Na^+^, operative time, anhepatic phase, induction regimen, and ICU stay as significant predictors. The SVM model demonstrated superior predictive performance, with area under the curve values of 0.881 (95% CI: 0.83–0.93) and 0.786 (95% CI: 0.69–0.88) in the training and test sets, respectively. The calibration curve showed high agreement between the predicted and observed risks. The SVM model demonstrated superior specificity, sensitivity, F1 score, and recall compared to other models. SHAP analysis identified variables that contributed to the model predictions.

**Conclusions:**

This study constructed a robust predictive model for the perioperative co-occurrence of TCMR and pneumonia. The SVM model demonstrated superior predictive performance.

## Introduction

Liver transplantation (LT) has become the optimal treatment for end-stage liver disease. Although liver is considered an immunologically privileged organ among solid organ transplants (1), the incidence of acute rejection following LT can still reach 10–30% (2, 3). Theoretically, T-cell-mediated rejection (TCMR) typically occurs within 4 to 6 weeks post-transplantation, representing the period of most intense immune rejection in LT recipients (4). If not promptly intervened, it can lead to graft dysfunction and even graft failure (5). Although Banff classification criteria remain the gold standard for diagnosing TCMR (6), percutaneous liver biopsy, has limitations in the prompt identification. Concurrently, pneumonia is a common infectious complication during the perioperative period. It exacerbates hepatic ischemia-reperfusion injury, contributing to delayed graft function recovery (7), adversely impacting clinical outcomes of LT and posing a significant threat to recipient survival (8). Hence, it is critically urgent to refine prognostic tools. Precise risk stratification for TCMR and pneumonia occurrence is essential for optimizing perioperative care. This may provide a framework for elucidating the pathophysiological mechanisms of perioperative TCMR and pneumonia in LT and developing individualized dynamic surveillance protocols. Such advancements hold significant implications for enhancing patient quality of life and prolonging survival outcomes, thereby optimizing long-term prognosis in this high-risk population. Postoperative pneumonia risk stratification has been enhanced by recently developed scoring systems, including the ISAN score (Intracerebral Hemorrhage, Sex, Age, NIH Stroke Scale) (9), Pneumonia Risk Index (non-cardiac surgery) (10), and Systemic Inflammation Score (post-gastrectomy) (11). However, these scoring systems are not applicable for assessing pneumonia after LT. They fail to account for the multifactorial synergy driving the co-occurrence of TCMR and pneumonia and struggle to capture nonlinear relationships or higher-order interactions among variables. Furthermore, conventional models exhibit low sensitivity in predicting TCMR post-LT (12), making it difficult to optimize immunosuppressive regimens for individual patients. For instance, while pulse steroid therapy can reduce rejection risk, it increases infection probability; traditional models are inadequate for quantifying such dynamic trade-offs.

In contrast, machine learning (ML) algorithms—through automated feature extraction and nonlinear modeling—demonstrate superior prediction accuracy. Techniques such as gradient-boosted ensembles outperform conventional scoring systems in predicting perioperative TCMR and pneumonia occurrence by decoding complex clinical variable interactions. Compared to traditional scoring systems, ML models have shown promising performance in predicting outcomes across various solid organ transplantation (13, 14). Currently, ML is widely applied to predict outcomes after LT (15, 16). Chen et al. (17) developed an ML model to predict pneumonia occurrence after LT. Maryam et al. (18) developed and validated an ML model demonstrating good performance in predicting plasma cell-rich rejection after LT. To mitigate the incidence of rejection following LT, Yoon et al. (19) employed ML algorithms to predict the optimal therapeutic range of tacrolimus, thereby advancing the clinical implementation of personalized immunosuppressive regimens. However, ML models specifically predicting the co-occurrence of perioperative TCMR and pneumonia after LT are scarce.

This study aims to develop and validate an ML-based predictive model for the co-occurrence of perioperative TCMR and pneumonia after LT. Ensemble algorithms and SHapley Additive exPlanations interpretability (SHAP) will be employed to identify nonlinear interactions and latent risk patterns that conventional methods may overlook, thereby addressing critical gaps in current prognostic frameworks. The clinical significance of this study lies in its potential to transform perioperative management strategies. With precise risk stratification, the established model could guide personalized therapeutic interventions, optimize surveillance protocols, inform immunosuppression regimen adjustments, and optimize the rational use of antimicrobial agents. These advances hold promise for reducing serious complications arising from perioperative TCMR and pneumonia after LT, thereby providing a basis for clinical decision-making.

## Methods

### Study cohort

This retrospective study obtained clinical data from LT patients at the Institute of Transplantation Medicine, Second Affiliated Hospital of Guangxi Medical University, between November 1, 2019, and June 1, 2025. All allografts originated from deceased donors, with allocation governed by the China Organ Transplant Response System. The study was conducted in accordance with both the Declarations of Helsinki and Istanbul and the study protocol was ratified by the Institutional Review Board of the hospital (Approval No.: 2019-(KY-0113)), and all participants provided informed consent. Inclusion criteria encompassed: (1) age ≥18 years at the time of primary deceased-donor allogeneic LT; (2) absence of active infection at transplantation: negative blood cultures within 48 hours preoperatively and no radiological evidence of infection; (3) normal preoperative immune status: CD4+ T-cell count ≥200/μL; (4) TCMR meeting either criterion: histologically confirmed per Banff 2023 criteria (biopsy-proven); clinical diagnosis: ALT/AST elevation ≥3× baseline and FK506 <5 ng/mL; (5) pneumonia diagnosis requiring: radiological confirmation and/or microbiological evidence; (6) co-occurrence requirement: TCMR and pneumonia diagnoses within the perioperative period and interval between TCMR and pneumonia ≤7 days; (7) availability of standardized postoperative follow-up data. Exclusion criteria encompassed: (1) combined multi-organ transplantation; (2) pre-existing structural lung disease or chronic respiratory failure; (3) active systemic infection at transplantation; (4) mortality or retransplantation within perioperative period (excluding cases caused by TCMR and pneumonia); (5) ABO-incompatible LT. In this study, the perioperative period was defined as 30 days after LT.

### Data collection

The following information was retrospectively collected: (1) demographic parameters: gender; age; body mass index (BMI); blood type; (2) preoperative laboratory parameters: white blood cell (WBC) count; hemoglobin (Hb); platelet (PLT) count; neutrophil (NEUT) count; lymphocyte (LYM) count; creatine kinase (CK); creatine kinase-MB isoenzyme (CK-MB); procalcitonin (PCT); C-reactive protein (CRP); total cholesterol (TC); triglyceride (TG); high-density lipoprotein (HDL); low-density lipoprotein (LDL); serum creatinine (SCr); blood urea nitrogen (BUN); uric acid (UA); cystatin C (Cys C); CD4+ T-lymphocyte (CD4+) count; CD8+ T-lymphocyte (CD8+) count; B cell (BC) count; natural killer cell (NK) count; K^+^; Na^+^; Cl^+^; Ca^2+^; Mg^2+^; PH; PO_2_; PCO_2_; A-aDO_2_; (3) preoperative concurrent symptoms: hepatic encephalopathy; ascites; (4) postoperative laboratory parameters (postoperative day 7): postoperative total bilirubin (TBIL); postoperative direct bilirubin (DBIL); postoperative albumin (ALB); postoperative gamma-glutamyl transpeptidase (GGT); postoperative aspartate aminotransferase (AST); postoperative alanine aminotransferase (ALT); postoperative alkaline phosphatase (ALP); postoperative prothrombin time (PT); postoperative international normalized ratio (INR); postoperative activated partial thromboplastin time (APTT); postoperative CD4+; postoperative CD8+; postoperative BC; postoperative NK; postoperative PH; postoperative PO_2_; postoperative PCO_2_; postoperative A-aDO_2_; (5) surgical metrics: operation time; anhepatic phase; blood loss; packed red blood cells (PRBC); fresh frozen plasma (FFP); apheresis platelets; mechanical ventilation (MV); ICU stay; (6) immunosuppressive management: human leukocyte antigen class I antibody (HLA-I Ab); human leukocyte antigen class II antibody (HLA-II Ab); induction therapy; immunotherapy regimen; tacrolimus (FK506); (7) donor gender; donor age; donor BMI; (8) gender matched.

### Statistical analysis

All data were processed and visualized in the R statistical computing environment 4.4.0. The ggplot2 package was utilized for graphical representations. Two-tailed analyses were utilized for hypothesis testing, with P<0.05 implying statistical significance. The normality of continuous variables was determined via the Shapiro-Wilk test. Variables in normal distribution were depicted as mean ± standard deviation (SD), while non-normally distributed variables were depicted as median (interquartile range [IQR]) and compared with the Mann-Whitney U test. Categorical data were reported as frequencies (percentages), and pairwise comparisons were performed via Pearson’s χ² test or Fisher’s exact test, as appropriate. For variables exceeding the predetermined 20% missingness threshold, imputation was performed using the Random Forest algorithm within the Multivariate Imputation by Chained Equations (MICE) package (v3.16.2).

### Feature variable screening

LASSO regression identified significant predictors between clinical characteristics of LT recipients and the perioperative co-occurrence of TCMR and pneumonia following LT. The iterative analysis was conducted via a 10-fold cross-validation method, λ-min (minimum lambda): 0.01906806. Variables with statistical significance (*P* < 0.05) in both LASSO regression and comparative analyses were selected for multivariate modeling. These variables were subsequently incorporated into predictive model development.

### Development and evaluation of predictive models

Predictive models were constructed for perioperative co-occurrence of TCMR and pneumonia after LT (binary outcome: 1=co-occurrence, 0=no co-occurrence). Five classical ML algorithms were implemented: logistic regression (LR), support vector machine (SVM), random forest (RF), gradient boosting machine (GBM), and extreme gradient boosting (XGBoost). The cohort was randomly stratified into training (70%) and test (30%) sets.

Receiver operating characteristic (ROC) curves assessed model performance, with the AUC and 95% CI reckoned to quantify discrimination accuracy. The optimal diagnostic cutoff was identified by the Youden index (J=sensitivity + specificity – 1), from which corresponding sensitivity and specificity values were derived. The calibration curve was implemented to estimate high agreement between the predicted and observed risks. Model performance was additionally evaluated using specificity, sensitivity, F1-score, and recall rate. Comparative analysis of these metrics across all algorithms was made to identify the optimal model for subsequent clinical assessment.

### Interpretability analysis

A swarm plot was created using the SHAP method to present the individual contribution of each feature to the prediction. SHAP evaluations revealed the degree to which each feature influenced specific samples, thereby elucidating the model’s decision-making procedures. Ultimately, feature recursive elimination was utilized to screen variables to simplify the model.

## Results

### Clinical characteristics

This study enrolled 717 patients who underwent LT, including 600 males (83.7%) and 117 females (16.3%). Based on perioperative co-occurrence of TCMR and pneumonia after LT, patients were allocated into a co-occurrence group (93 cases) and a no co-occurrence group (624 cases). Biopsy-proven TCMR: 56 cases (accounting for 60.2% of total TCMR cases); clinically diagnosed TCMR: 37 cases (accounting for 39.8% of total TCMR cases). Demographic and clinical traits are listed in **Table 1**. Most variables showed comparable distributions between training and test sets (*P* > 0.05). However, the following variables demonstrated statistically significant differences (*p* < 0.05) between the training and testing cohorts: blood type, WBC, PLT, NEUT, TG, UA, CysC, CD8+, and Mg^2+^.

**Table 1:** Clinical characteristics of recipients.

Variable	All	Training set	Test set	P value
	(n=717)	(n=501)	(n=216)	
Gender (n, %)				0.349
Male	600 (83.7%)	424 (84.6%)	176 (81.5%)	
Female	117 (16.3%)	77 (15.4%)	40 (18.5%)	
Age (years)	51.0 [43.0;57.0]	51.0 [43.0;56.0]	50.0 [42.0;57.2]	0.707
Blood type (n, %)				0.021
A	169 (23.6%)	118 (23.6%)	51 (23.6%)	
B	169 (23.6%)	103 (20.6%)	66 (30.6%)	
O	42 (5.86%)	33 (6.59%)	9 (4.17%)	
AB	337 (47.0%)	247 (49.3%)	90 (41.7%)	
BMI (kg/m²)	23.0 [20.9;25.3]	23.1 [21.0;25.2]	22.9 [20.8;25.5]	0.891
WBC (10^9^/L)	4.56 [3.30;6.53]	4.79 [3.40;6.56]	4.10 [3.01;6.38]	0.013
Hb (g/L)	101 [81.0;123]	102 [82.0;124]	100 [78.0;120]	0.31
PLT (10^9^/L)	80.0 [50.0;130]	81.0 [51.5;141]	75.5 [43.0;113]	0.02
NEUT(10^9^/L)	3.03 [1.98;4.57]	3.14 [2.08;4.67]	2.62 [1.85;4.36]	0.013
LYM(10^9^/L)	0.85 [0.57;1.24]	0.86 [0.58;1.25]	0.82 [0.53;1.23]	0.352
CK (u/L)	77.0 [46.0;124]	79.0 [46.0;127]	71.5 [44.8;115]	0.214
CKMB (u/L)	30.0 [17.0;48.0]	30.0 [17.0;48.0]	29.0 [16.0;48.0]	0.918
PCT (ng/mL)	0.16 [0.07;0.44]	0.17 [0.08;0.44]	0.15 [0.07;0.42]	0.22
CRP (mg/L)	7.78 [2.65;21.2]	8.08 [2.76;22.9]	6.42 [2.45;17.8]	0.127
TC (mmol/L)	3.10 [2.19;4.09]	3.05 [2.19;4.16]	3.12 [2.20;3.88]	0.635
TG (mmol/L)	0.90 [0.68;1.30]	0.88 [0.66;1.24]	0.96 [0.79;1.53]	0.007
HDL (mmol/L)	0.89 [0.50;1.23]	0.90 [0.48;1.26]	0.88 [0.52;1.18]	0.475
LDL (mmol/L)	1.77 [1.18;2.53]	1.83 [1.17;2.63]	1.69 [1.21;2.39]	0.162
SCr (μmol/L)	78.0 [64.0;101]	78.0 [64.0;100]	79.0 [65.0;101]	0.455
BUN (mmol/L)	5.13 [3.85;7.51]	5.09 [3.76;7.22]	5.22 [4.03;7.93]	0.297
UA (μmol/L)	283 [209;378]	272 [202;370]	302 [220;393]	0.012
CysC (mg/L)	1.19 [0.95;1.60]	1.18 [0.92;1.57]	1.27 [1.00;1.70]	0.028
CD4+ (μL)	314 [176;497]	315 [179;510]	304 [172;482]	0.435
CD8+ (μL)	162 [87.0;274]	173 [95.0;280]	145 [75.8;258]	0.017
BC (μL)	131 [70.0;210]	137 [76.0;219]	112 [64.8;197]	0.066
NK (μL)	76.0 [40.0;151]	80.0 [41.0;159]	67.0 [39.0;134]	0.158
K^+^ (mmol/L)	3.83 [3.57;4.12]	3.81 [3.55;4.11]	3.90 [3.64;4.13]	0.1
Na^+^ (mmol/L)	138 [135;140]	138 [135;140]	138 [135;140]	0.862
Cl^+^ (mmol/L)	105 [102;108]	105 [102;108]	106 [102;109]	0.202
Ca^2+^ (mmol/L)	2.15 [2.05;2.25]	2.14 [2.05;2.25]	2.16 [2.06;2.27]	0.368
Mg^2+^ (mmol/L)	0.81 [0.75;0.88]	0.82 [0.76;0.88]	0.80 [0.72;0.87]	0.035
PH	7.43 [7.40;7.46]	7.43 [7.40;7.46]	7.43 [7.40;7.46]	0.86
PO_2_ (mmHg)	93.8 [80.0;107]	94.2 [81.0;108]	93.0 [78.7;106]	0.394
PCO_2_(mmHg)	34.9 [31.1;38.2]	34.9 [31.1;38.7]	34.9 [31.2;37.8]	0.522
A-aDO_2_(mmHg)	28.0 [14.6;60.2]	27.9 [13.6;60.2]	28.1 [15.6;59.7]	0.411
Operation time (minutes)	443 [400;506]	444 [399;505]	442 [403;514]	0.74
Anhepatic phase (minutes)	40.0 [35.0;48.0]	41.0 [35.0;48.0]	40.0 [35.8;47.0]	0.389
Blood loss (mL)	500 [400;800]	500 [400;800]	500 [400;800]	0.744
PRBC (u)	4.00 [0.00;7.50]	4.00 [0.00;7.00]	4.00 [0.00;8.00]	0.141
FFP (mL)	610 [0.00;1020]	610 [0.00;1010]	625 [0.00;1050]	0.2
Apheresis platelets (u)	0.00 [0.00;1.00]	0.00 [0.00;1.00]	0.00 [0.00;1.00]	0.667
MV (hours)	12.5 [9.00;20.0]	12.0 [9.00;20.0]	13.0 [9.00;23.0]	0.206
ICU stay (hours)	163 [139;193]	162 [139;190]	164 [138;209]	0.859
FK506 (ng/mL)	4.00 [2.50;5.70]	4.00 [2.60;5.80]	3.85 [2.48;5.40]	0.126
Postop-TBIL (μmol/L)	35.1 [15.0;133]	34.4 [14.7;131]	36.2 [15.4;142]	0.554
Postop-DBIL (μmol/L)	18.0 [7.30;88.7]	17.5 [6.90;88.7]	19.2 [8.00;87.8]	0.51
Postop-ALB (g/L)	33.9 [29.9;37.7]	34.0 [30.0;38.0]	33.2 [29.1;37.3]	0.152
Postop-GGT (u/L)	60.0 [31.0;123]	62.0 [31.0;121]	57.5 [31.8;125]	0.737
Postop-AST (u/L)	50.0 [33.0;88.0]	52.0 [33.0;90.0]	45.0 [32.0;82.0]	0.11
Postop-ALT (u/L)	33.0 [20.0;56.0]	35.0 [22.0;57.0]	30.0 [19.0;55.0]	0.098
Postop-ALP (u/L)	129 [96.0;189]	128 [96.0;184]	132 [97.5;198]	0.439
Postop-PT (seconds)	15.2 [13.0;19.4]	15.1 [12.9;19.5]	15.6 [13.1;19.0]	0.632
Postop-INR	1.38 [1.17;1.76]	1.37 [1.17;1.77]	1.40 [1.18;1.74]	0.677
Postop-APTT (seconds)	35.3 [32.0;40.7]	35.3 [31.8;40.8]	35.4 [32.1;40.5]	0.74
Postop-CD4+ (μL)	121 [61.0;252]	123 [59.0;246]	115 [62.0;258]	0.74
Postop-CD8+ (μL)	81.0 [38.0;153]	82.0 [38.0;156]	80.0 [36.8;146]	0.436
Postop-BC (μL)	128 [63.0;242]	128 [64.0;243]	127 [62.0;224]	0.603
Postop-NK (μL)	37.0 [18.0;73.0]	37.0 [17.0;74.0]	38.0 [20.0;68.2]	0.871
Postop-PH	7.41 [7.38;7.44]	7.41 [7.38;7.44]	7.41 [7.38;7.45]	0.467
Postop-PO_2_(mmHg)	106 [79.9;142]	107 [83.5;141]	101 [77.6;144]	0.568
Postop-PCO_2_(mmHg)	39.1 [35.9;42.8]	39.2 [35.9;43.0]	39.0 [36.0;42.0]	0.589
Postop-A-aDO_2_(mmHg)	94.5 [49.1;140]	92.0 [48.4;136]	103 [50.6;151]	0.276
HLA-IAb (n, %)				0.15
No	690 (96.2%)	486 (97.0%)	204 (94.4%)	
Yes	27 (3.77%)	15 (2.99%)	12 (5.56%)	
HLA-IIAb (n, %)				0.771
No	691 (96.4%)	484 (96.6%)	207 (95.8%)	
Yes	26 (3.63%)	17 (3.39%)	9 (4.17%)	
Induction regimen (n, %)				0.782
No	236 (32.9%)	167 (33.3%)	69 (31.9%)	
Yes	481 (67.1%)	334 (66.7%)	147 (68.1%)	
Immunotherapy regimen (n, %)				0.403
Pred+Tac+MMF	702 (97.9%)	492 (98.2%)	210 (97.2%)	
Pred+Tac+others	15 (2.09%)	9 (1.80%)	6 (2.78%)	
Ascites (n, %)				0.297
No	483 (67.4%)	344 (68.7%)	139 (64.4%)	
Yes	234 (32.6%)	157 (31.3%)	77 (35.6%)	
Hepatic encephalopathy (n, %)				0.263
No	646 (90.1%)	456 (91.0%)	190 (88.0%)	
Yes	71 (9.90%)	45 (8.98%)	26 (12.0%)	
Donor age (years)	44.3 [26.8;52.3]	44.0 [26.2;52.7]	44.7 [26.5;52.6]	0.637
Donor gender (n, %)				0.452
Male	504 (70.3%)	378 (69.1%)	116 (72.5%)	
Female	213 (29.7%)	169 (30.9%)	44 (27.5%)	
Donor BMI (kg/m²)	23.5 [20.4;25.1]	23.2 [20.8;25.5]	23.6 [20.8;25.6]	0.217
Gender matched (n, %)				0.513
No	118 (16.5%)	85 (17.0%)	33 (15.2%)	
Yes	599 (83.5%)	415 (83.0%)	184 (84.8%)	
ABO incompatibility (n, %)	0	0	0	0

BMI, body mass index; TBIL, total bilirubin; DBIL, direct bilirubin; ALB, albumin; GGT, gamma-glutamyl transpeptidase; AST, aspartate aminotransferase; ALT, alanine aminotransferase; ALP, alkaline phosphatase; PT, prothrombin time; INR, international normalized ratio; APTT, activated partial thromboplastin time; WBC, white blood cell; Hb, hemoglobin; PLT, platelet; NEUT, neutrophil; LYM, lymphocyte; CK, creatine kinase; CKMB, creatine kinase-MB Isoenzyme; PCT, procalcitonin; CRP, C-reactive protein; TC, total cholesterol; TG, triglyceride; HDL, high-density lipoprotein; LDL, low-density lipoprotein; SCr, serum creatinine; BUN, blood urea nitrogen; UA, uric acid; CysC, cystatin C; CD4+, CD4+ T-lymphocyte; CD8, CD8+ T-lymphocyte; BC, B cell; NK, natural killer cell; PRBC, packed red blood cells; FFP, fresh frozen plasma; MV, mechanical ventilation; HLA -IAb, human leukocyte antigen class I antibody; HLA -IIAb: human leukocyte antigen class II antibody; Postop, Postoperative.

### Feature selection

**Figure 1:**
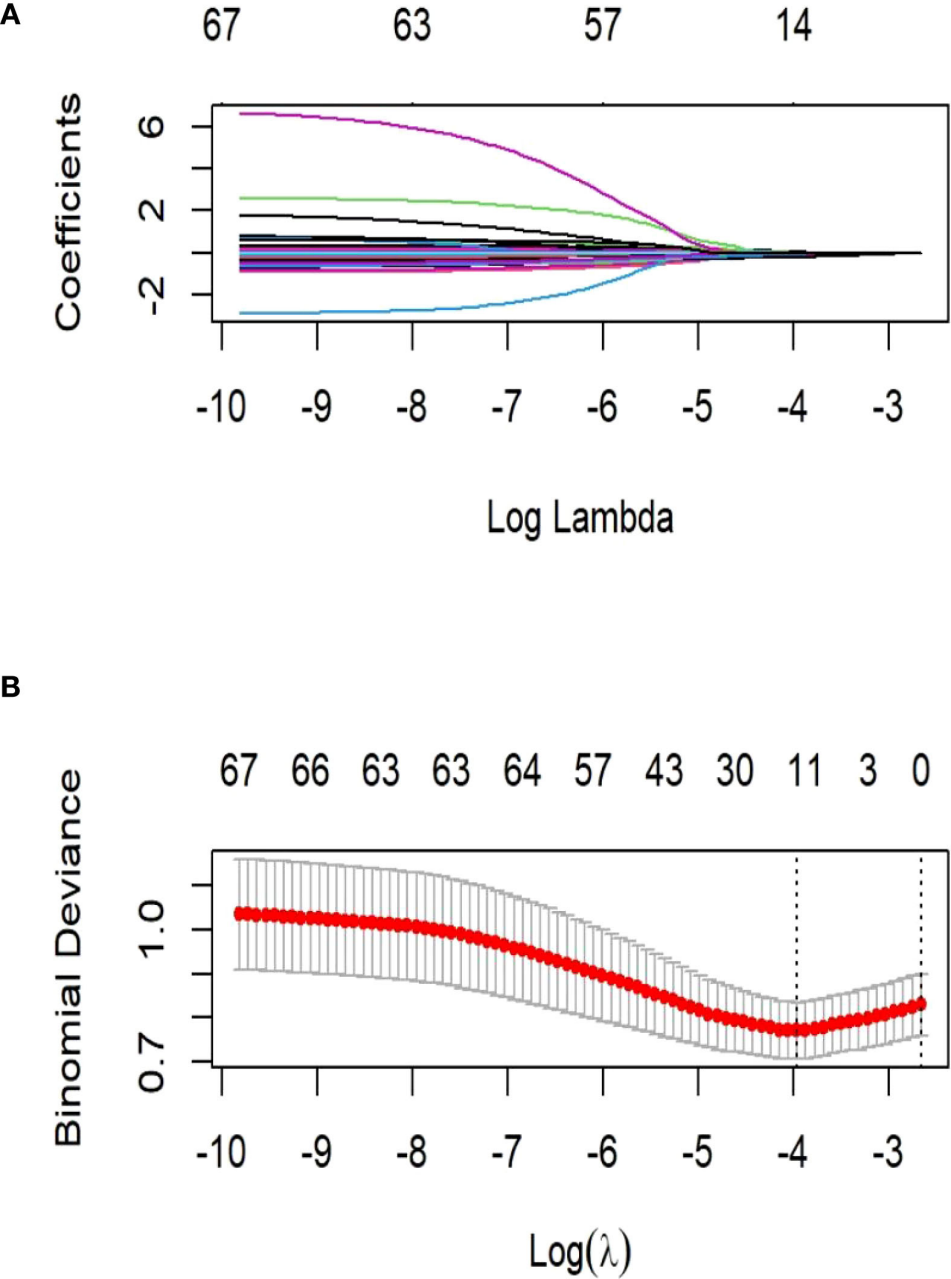
Demographic and clinical feature selection using the least absolute shrinkage and selection operator (LASSO) binary logistic regression model. **(A)** LASSO coefficient profiles of the 21 features. A coefficient profile plot was produced against the log (λ) sequence. The vertical line was drawn at the value selected using 10-fold cross-validation, where optimal resulted in 6 features with non-zero coefficients. **(B)** Tuning parameter (λ) selection in the LASSO model used 10-fold cross-validation via minimum criteria. The partial likelihood deviance (binomial deviance) curve was plotted versus log(λ).

LASSO regression analysis in the training cohort (70% of the total sample) identified postoperative DBIL, postoperative INR, HDL, postoperative ALT, NK, FK506, NA^+^, operative time, anhepatic phase, induction regimen, and ICU stay as significant predictors of perioperative co-occurrence TCMR and pneumonia (**Figures 1A, B**). These variables were subsequently integrated into ML algorithms to establish a robust prediction model.

### Model performance assessment

**Figure 2:**
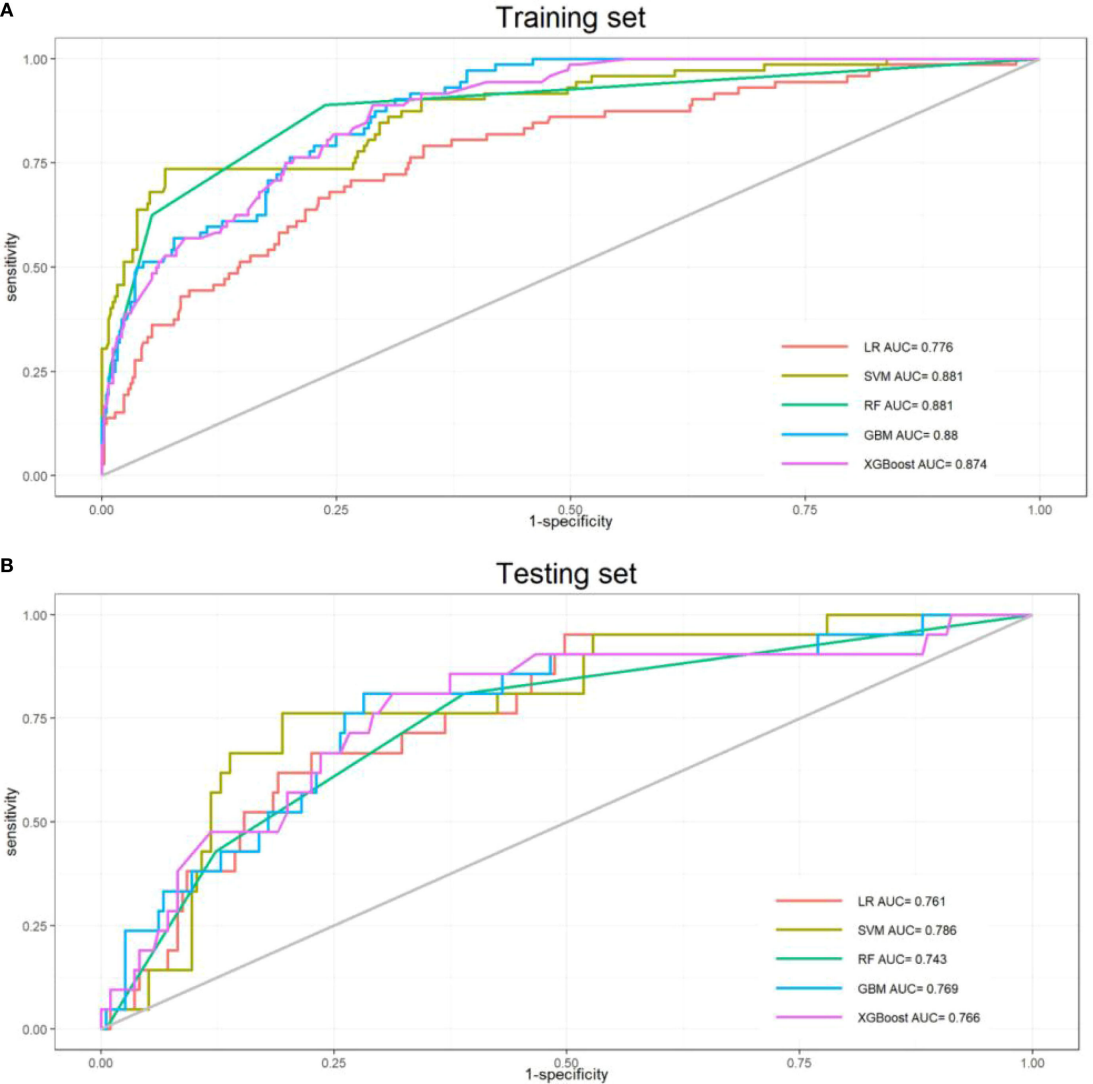
Receiver operating characteristic curves for the five models. **(A)**Training sets; **(B)** Test sets).

**Figure 3:**
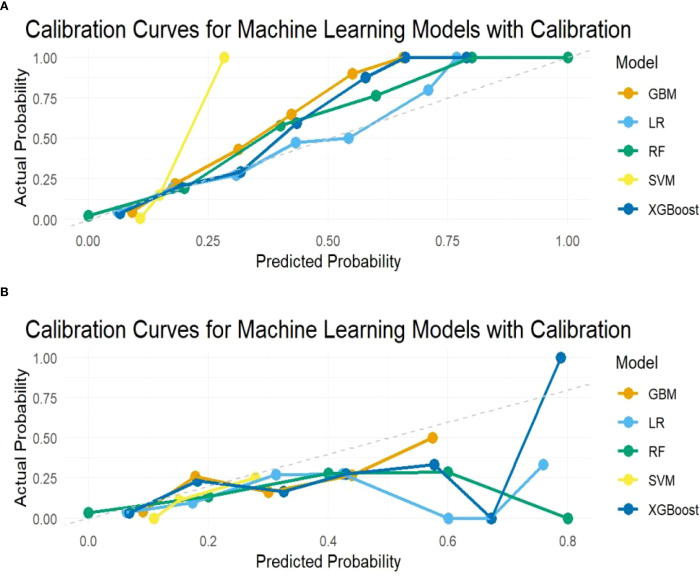
Calibration curves of the predicted probability. **(A)** Training sets; **(B)** Test sets.

To determine the optimal model for predicting perioperative co-occurrence TCMR and pneumonia, five distinct algorithms were compared. The predictive power of these models was comprehensively evaluated via ROC curves and AUC values. Results demonstrated that the SVM model consistently achieved superior AUC values of 0.881 (95% CI: 0.83–0.93) in the training dataset and 0.786 (95% CI: 0.69–0.88) in the test dataset, outperforming all other models (**Figures 2A, B**). Furthermore, additional binary classification metrics—including AUC, sensitivity, recall, specificity, accuracy, precision, and F1-score—were evaluated (**Table 2**). The SVM model exhibited statistically significant advantages across these metrics compared to alternative models (**Figures 3A, B**), further validating its predictive capability for co-occurrence TCMR and pneumonia.

**Table 2:** Evaluating the predictive performance of each model.

Model	AUC		Specificity		Sensitivity		Accuracy		Precision		Recall		F1-score	
	Training	Test	Training	Test	Training	Test	Training	Test	Training	Test	Training	Test	Training	Test
LR	0.776 (95% CI: 0.72-0.84)	0.761 (95% CI: 0.66-0.86)	0.657	0.503	0.792	0.952	0.677	0.546	0.280	0.171	0.792	0.952	0.414	0.290
SVM	0.881 (95% CI: 0.83-0.93)	0.786 (95% CI: 0.69-0.88)	0.932	0.805	0.736	0.762	0.904	0.801	0.646	0.296	0.736	0.762	0.668	0.426
RF	0.881 (95% CI: 0.84-0.93)	0.743 (95% CI: 0.64-0.85)	0.762	0.610	0.889	0.810	0.780	0.630	0.386	0.183	0.889	0.810	0.538	0.299
GBM	0.880 (95% CI: 0.85-0.92)	0.769 (95% CI: 0.66-0.88)	0.688	0.718	0.903	0.810	0.719	0.727	0.327	0.236	0.903	0.810	0.480	0.366
XGBoost	0.874 (95% CI: 0.84-0.91)	0.766 (95% CI: 0.65-0.88)	0.711	0.687	0.889	0.810	0.737	0.699	0.340	0.218	0.889	0.810	0.492	0.344

LR, logistic regression; SVM, support vector machine; RF, random forest; GBM, gradient boosting machine; XGBoost, extreme gradient boosting.

### Interpretability analysis

**Figure 4:**
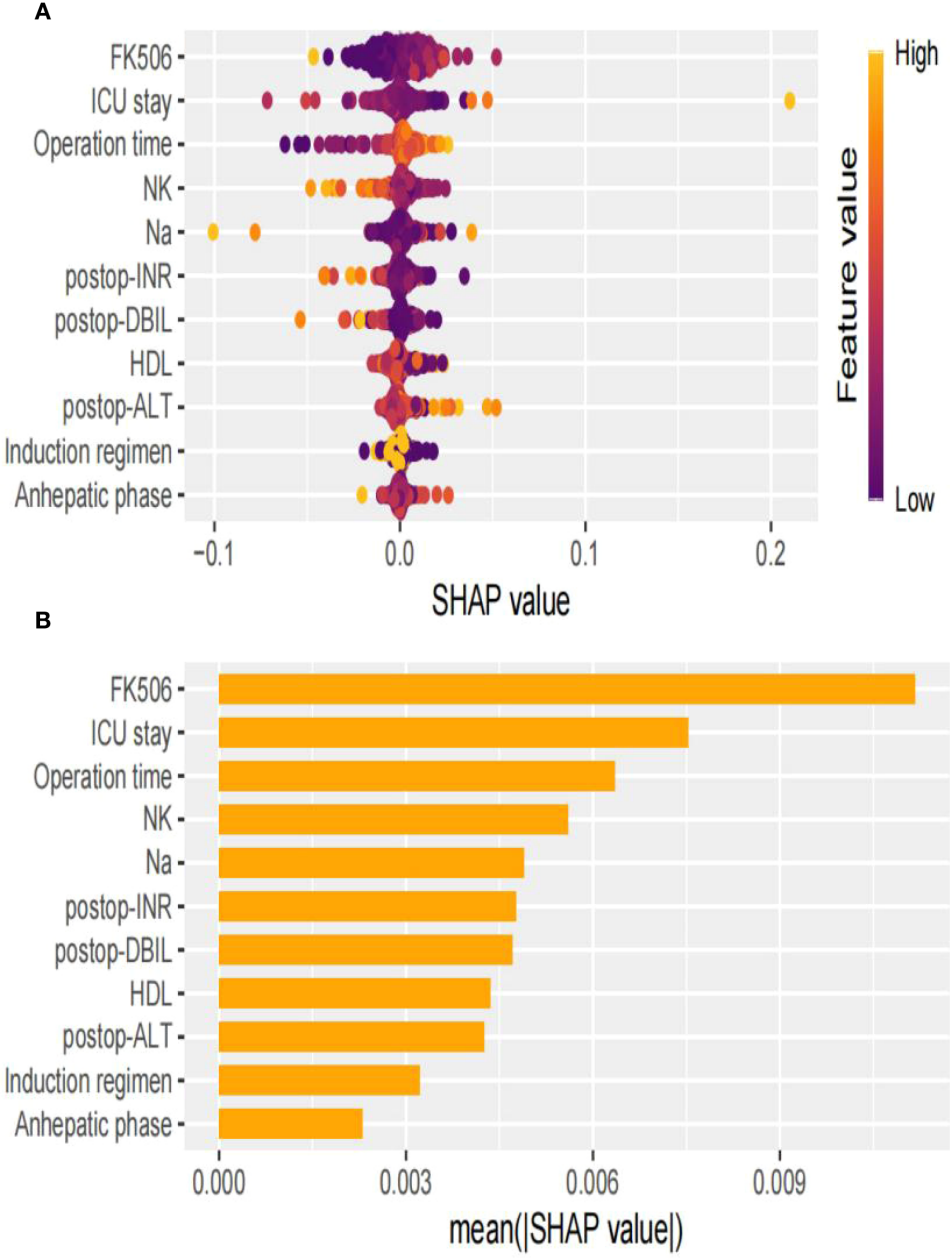
Visual explanation of perioperative co-occurrence of TCMR and pneumonia model based on SVM. **(A)** The SHapley Additive explanation; **(B)** Feature importance scores.

**Figure 5:**
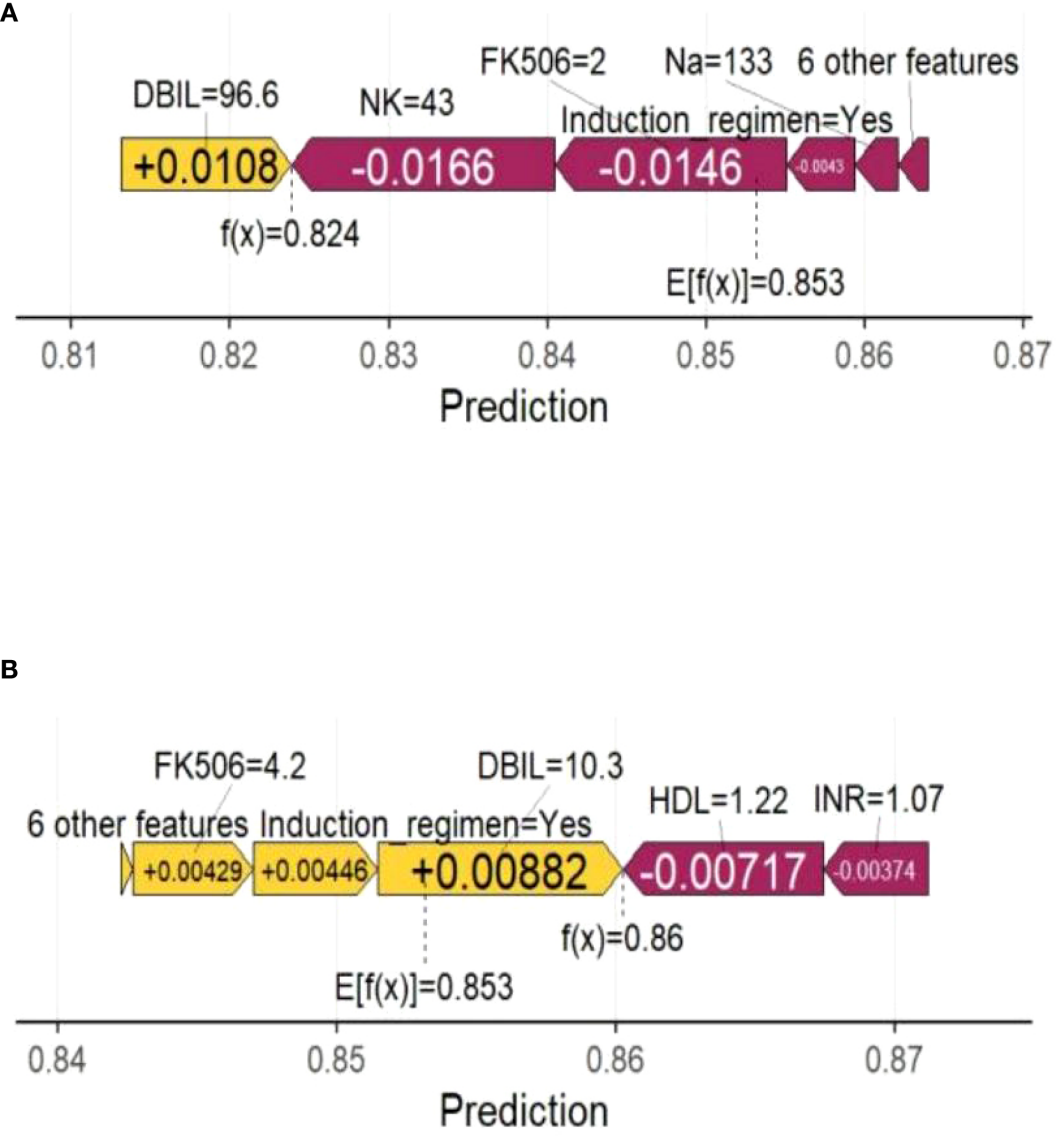
Force plot shows the contribution of each feature to the prediction result of using the SVM model. **(A)** One sample with a positive outcome prediction; **(B)** One sample with a negative outcome prediction. Orange bars indicate features that contribute positively to the prediction, while purple bars indicate negative contributions. Feature values are shown alongside their SHAP values.

In the swarm diagram (**Figure 4A**), the horizontal axis was SHAP values, and the vertical axis demonstrated features. Each data point reflected a specific instance, with its position on the x-axis representing the SHAP value for a particular feature. The analysis identified FK506, ICU stay, operation time, and NK cell as the four most influential predictors (**Figures 4A, B**). Notably, NK cell exerted a negative effect on perioperative co-occurrence TCMR and pneumonia. To demonstrate the SHAP calculation process, representative samples were selected: one with a positive outcome prediction (**Figure 5A**) and one with a negative outcome prediction (**Figure 5B**). The SVM-derived SHAP plot illustrates feature contributions for two patients. Orange/purple bars denote positive/negative impacts, with actual values alongside SHAP values.

## Discussion

Accurate prediction of risk factors for perioperative co-occurrence TCMR and pneumonia following LT is critical for timely intervention and improved outcomes. This study focused on recipients undergoing LT and validated a predictive model for assessing the simultaneous occurrence of TCMR and pneumonia during the perioperative period. The model integrated preoperative clinical characteristics, immunological interventions, dynamic changes in surgical parameters, preoperative concurrent symptoms, postoperative clinical characteristics, and donor information utilizing multiple ML algorithms. Comparative analysis of ML models revealed that the SVM algorithm achieved the optimal predictive accuracy, with AUC values of 0.881 (95% CI: 0.83–0.93) in the training set and 0.786 (95% CI: 0.69–0.88) in the test set. Calibration demonstrated high predicted-observed risk concordance. Furthermore, the SVM model demonstrated a high specificity, sensitivity, and F1 score. These findings indicate that ML models incorporating recipient-specific multidimensional data can effectively stratify perioperative co-occurrence TCMR and pneumonia, offering actionable insights for optimizing clinical decision-making and postoperative management.

The comparative analysis demonstrated the substantial advantage of ML models in prediction tasks (15, 20, 21). Torres et al. developed a machine learning-based model (K-prototype clustering algorithm) to predict post-liver transplant complications including acute rejection and infection, demonstrating favorable performance (22). This advantage can be attributable to ML’s ability to cope with complex data through advanced regularization techniques and ensemble learning mechanisms (23). Particularly, ML exhibits higher accuracy in capturing non-linear relationships, suggesting that statistical models may simplify complex biomedical interactions. Previous studies predominantly rely on clinical experience for variable selection, have confirmed the utility of ML in predicting TCMR or pneumonia prediction (17, 18). In contrast, this study integrated preoperative and postoperative clinical characteristics, immunological interventions, preoperative concurrent symptoms, donor information, and dynamic changes in surgical procedures to objectively identify key predictors (postoperative DBIL, postoperative INR, postoperative ALT, HDL, NK, FK506, Na^+^, operative time, anhepatic phase, induction regimen, and ICU stay) through LASSO regression. These predictors originated from objective data. A ML-based predictive model for perioperative co-occurrence TCMR and pneumonia following LT was subsequently developed.

As the most critical predictor in this model, the blood concentration of FK506 is paramount for preventing graft rejection and reducing the risk of infection, particularly pneumonia (24). A delicate therapeutic balance exists between these competing outcomes. FK506 suppresses the cellular immune response against the hepatic allograft primarily by inhibiting T-lymphocyte activation and proliferation, achieved through blockade of key cytokine transcription, including interleukin-2 (25). Subtherapeutic concentrations result in insufficient immunosuppression, failing to adequately inhibit recipient T-cell recognition and attack of the donor liver. This predisposes patients to acute cellular rejection, manifested by abnormal liver function tests (elevated transaminases and bilirubin). Severe or recurrent rejection episodes can lead to graft dysfunction or loss (5). Conversely, supratherapeutic concentrations, while theoretically offering enhanced rejection prophylaxis, incur significant costs: a marked increase in drug toxicity (26) and the risk of severe infections (27). Consequently, indiscriminately maintaining excessively high concentrations solely to achieve “absolute rejection avoidance” is clinically contraindicated, as the associated risks substantially outweigh potential benefits. FK506 exhibits a narrow therapeutic index between effective immunosuppression (preventing rejection) and toxic concentrations (predisposing to infection/toxicity) (28). Furthermore, significant interindividual variability in blood concentrations arises due to factors influencing drug metabolism, including genetics (e.g., CYP3A5 polymorphisms), age, hepatic and renal function, diet, and concomitant medications (29). Regular monitoring of FK506 trough levels is therefore fundamental. Based on these results, alongside individual rejection risk, infection susceptibility, and manifestations of drug toxicity, transplant clinicians must dynamically titrate the dosage through an individualized approach. The therapeutic goal is to maintain concentrations within a target range that effectively prevents rejection while minimizing the risks of infection and drug-related toxicity (27). ICU stay is also a significant predictor variable in our study. The duration of ICU hospitalization following LT serves as a critical indicator reflecting surgical complexity, graft functional recovery, severity of early complications, and the patient’s overall clinical status. While prolonged ICU stay itself is not a direct cause of rejection or pneumonia, it is strongly associated with and significantly increases the risk of developing both severe complications, with complex interactions existing between them. Prolonged ICU hospitalization is a well-established, highly significant independent risk factor for hospital-acquired pneumonia (30, 31). It is generally not the initiating cause of rejection; rather, severe rejection is often one of the primary factors leading to extended ICU stay. Furthermore, the complexity of the ICU environment and the patient’s critical condition significantly increase the difficulty of maintaining effective and stable immunosuppression, thereby indirectly elevating the risk of TCMR both during the ICU stay and shortly after transfer out of the ICU. Immunosuppression management poses substantial challenges in patients requiring protracted ICU care. In summary, the interplay between ICU length of stay, rejection, and pneumonia frequently establishes a vicious cycle. Severe rejection episodes often necessitate ICU admission and prolong hospitalization. The ICU environment and associated risk factors markedly increase the susceptibility to pneumonia (32). Severe pneumonia, in turn, necessitates reduction or discontinuation of immunosuppressive therapy to control the infection. Inadequate immunosuppression subsequently triggers rejection episodes or exacerbates existing rejection, thus creating a self-perpetuating cycle of adverse events. Operative time is also recognized as a significant predictive factor. Prolonged surgical time typically correlates with an extended anhepatic phase and increased total ischemic time. Sustained ischemia-reperfusion injury (IRI) leads to hepatic sinusoidal endothelial cell damage and microcirculatory disturbances, triggering the substantial release of damage-associated molecular patterns (33, 34). These damage-associated molecular patterns robustly activate the innate immune system, characterized by macrophage and neutrophil infiltration, and complement activation, resulting in the release of large quantities of pro-inflammatory cytokines (35). Damaged hepatocytes and endothelial cells exhibit upregulated expression of MHC molecules and co-stimulatory molecules (36), rendering them more recognizable as “non-self” by the recipient’s immune system. This facilitates the accelerated presentation of donor antigens to recipient T cells (37). Although the liver possesses inherent immunotolerance properties, severe IRI disrupts this microenvironment (38), significantly increasing the incidence and severity of early acute cellular rejection (39). Furthermore, prolonged operative duration is often associated with greater blood loss and substantial transfusion requirements. Allogeneic blood transfusion can induce complex immunomodulatory effects. It may also increase the risk of alloimmunization, including rejection directed against the graft, potentially through the introduction of allogeneic leukocyte antigens or by activating the recipient’s immune system (40). Extended operative time is a strong independent risk factor for postoperative pneumonia. A direct and well-established mechanistic link involves its association with longer durations of mechanical ventilation and an increased risk of pulmonary atelectasis (41). NK cells emerged as the fourth most important predictive variable in this study, exhibiting a negative regulatory role in the co-occurrence of TCMR and pneumonia. NK cells possess the ability to recognize and lyse virus-infected cells without requiring presensitization. They induce target cell apoptosis either through the release of perforin and granzymes or via the Fas ligand (FasL)/Fas pathway (42). Activated NK cells robustly secrete interferon-gamma and tumor necrosis factor-alpha. These cytokines not only exert direct antiviral effects (43) but also recruit neutrophils, monocytes/macrophages, and T cells to the site of infection, thereby amplifying the anti-infection immune response. Furthermore, NK cells can mediate antibody-dependent cellular cytotoxicity via FcγRIIIa (CD16a), enabling more effective killing of infected cells (44). In summary, insufficient NK cell counts can directly contribute to the development of perioperative pneumonia following LT. Viral infections themselves, particularly cytomegalovirus infection, constitute a significant risk factor for rejection (45). This creates a complex interplay: on the one hand, viral infection activates NK cells to combat the virus; on the other hand, cytokines like IFN-γ secreted by activated NK cells may simultaneously promote rejection. Conversely, the inflammation and tissue damage caused by rejection reactions also heighten the risk of infection.

This paper demonstrated that ML models could predict perioperative co-occurrence TCMR and pneumonia following LT. ML models exhibit superior abilities to capture complex, non-linear relationships and intricate interactions among clinical variables compared to traditional methods, thus offering valuable insights into individual patient risk profiles. This information can potentially inform personalized treatment strategies, optimize post-LT surveillance protocols, and ultimately improve patient outcomes.

### Clinical significance

The machine learning-based multidimensional prediction model (utilizing the SVM algorithm) developed and validated in this study holds paramount clinical value by providing a robust tool for the precise prevention and individualized management of TCMR-pneumonia comorbidity during the perioperative period following LT. Upon successful integration into the hospital’s electronic medical record (EMR) system as a clinical decision support system, this model empowers clinicians to implement preemptive alerts, accurate diagnosis, and early personalized interventions for high-risk patients. This effectively disrupts the vicious cycle of TCMR-pneumonia comorbidity, ultimately improving the prognosis of LT recipients and substantially alleviating familial and societal burdens. These outcomes demonstrate the transformative potential of AI-driven precision medicine in managing complex postoperative complications.

Real-time risk surveillance and early warning: the model’s core strength lies in its integration of critical, dynamically changing clinical indicators. Once deployed, the system automatically and continuously retrieves real-time data from the EMR, including: dynamic monitoring of FK506 blood concentration, dynamic assessment of immune cell function, dynamic evolution of liver function and coagulation parameters, surgical parameter retrospectives, and ICU length of stay. This dynamic data continuously updates risk assessments. Risk stratification based on predictive modeling: leveraging the integrated SVM algorithm, the system comprehensively analyzes the aforementioned real-time multidimensional data to compute an individualized risk score for current TCMR-pneumonia comorbidity occurrence. Patients are then automatically stratified into predefined risk tiers based on preset thresholds. Early identification and targeted intervention for high-risk patients: the explicit early-warning signals provided by the system offer clinicians precise diagnostic directionality. These alerts heighten clinical vigilance regarding potential comorbidity, prompting physicians to combine model outputs with specific patient presentations and necessary ancillary tests for accurate diagnosis at the disease’s earliest stage. Early diagnosis is prerequisite for effective intervention; for TCMR, warnings enable prompt judicious adjustment of immunosuppressive regimens.

### Limitations

There are also limitations. The single-center retrospective design may introduce selection bias, despite efforts to adjust for known confounders through multivariate analysis. Additionally, the primary limitation of this study is the absence of external validation on an independent, multi-center, large-scale cohort. This constraint impedes comprehensive assessment of our model’s generalizability at this stage and its immediate clinical applicability.

## Conclusions

This study developed a robust predictive model for perioperative co-occurrence TCMR and pneumonia in LT, and the SVM model achieved superior discriminative performance. Key predictors, including postoperative DBIL, postoperative INR, HDL, postoperative ALT, NK, FK506, Na^+^, operative time, anhepatic phase, induction regimen, and ICU stay, were identified as critical determinants of model. These results advance our understanding of the multifactorial pathogenesis of perioperative co-occurrence TCMR and pneumonia after LT, offering actionable insights for optimizing clinical decision-making and postoperative management.

## Data Availability

The original contributions presented in the study are included in the article/**Supplementary Material**. Further inquiries can be directed to the corresponding author.
