# Supplementary material for: Machine learning-based predictive model for the perioperative co-occurrence of T-cell-mediated rejection and pneumonia in liver transplantation
Source: Front Immunol. 2025 Sep 17;16:1648993. doi: 10.3389/fimmu.2025.1648993 (PMC12483920; doi:10.3389/fimmu.2025.1648993)
Supplement: Supplementary file 1 [file Table1.docx]

Supplementary 1. Missing rows

| Feature | Missing rows (%) |
| --- | --- |
| Gender | 0 |
| Age | 0 |
| Blood type | 0 |
| BMI | 2.23 |
| WBC | 0.14 |
| Hb | 0.14 |
| PLT | 0.14 |
| NEUT | 0.14 |
| LYM | 0.14 |
| CK | 0.7 |
| CKMB | 0.7 |
| PCT | 1.67 |
| CRP | 1.12 |
| TC | 8.79 |
| TG | 12.97 |
| HDL | 17.85 |
| LDL | 17.85 |
| SCr | 0.14 |
| BUN | 0.14 |
| UA | 0.14 |
| CysC | 0.14 |
| CD4+ | 6.56 |
| CD8+ | 6.56 |
| BC | 6.56 |
| NK | 6.56 |
| K^+^ | 0.42 |
| Na^+^ | 0.42 |
| Cl^+^ | 0.42 |
| Ca^2+^ | 0.42 |
| Mg^2+^ | 0.42 |
| PH | 8.09 |
| PO_2_ | 8.23 |
| PCO_2_ | 8.23 |
| A-aDO_2_ | 14.23 |
| Operation time | 0.56 |
| Anhepatic phase | 0.56 |
| Blood loss | 0.84 |
| PRBC | 2.23 |
| FFP | 2.23 |
| Apheresis platelets | 2.23 |
| MV | 0.84 |
| ICU stay | 1.12 |
| FK506 | 5.72 |
| Postop-TBIL | 0 |
| Postop-DBIL | 0 |
| Postop-ALB | 0 |
| Postop-GGT | 0 |
| Postop-AST | 0 |
| Postop-ALT | 0 |
| Postop-ALP | 0 |
| Postop-PT | 0 |
| Postop-INR | 0 |
| Postop-APTT | 0 |
| Postop-CD4+ | 5.58 |
| Postop-CD8+ | 5.58 |
| Postop-BC | 5.58 |
| Postop-NK | 5.58 |
| Postop-PH | 7.95 |
| Postop-PO_2_ | 7.95 |
| Postop-PCO_2_ | 7.95 |
| Postop-A-aDO_2_ | 7.95 |
| HLA-ⅠAb | 0 |
| HLA-ⅡAb | 0 |
| Induction regimen | 0 |
| Immunotherapy regimen | 0 |
| Ascites | 0 |
| Hepatic encephalopathy | 0 |
| Donor age | 0 |
| Donor gender | 0 |
| Donor BMI | 0 |
| Gender matched | 0 |

| Models | Parameter |
| --- | --- |
| SVM | cost = 150, gamma = 0.01 |
| RF | Ntree = 5, mtry= 1, nodesize = 10 |
| GBM | distribution = "bernoulli", data = train_data, n.trees = 300, shrinkage = 0.005, interaction.depth = 2, n.minobsinnode = 3, cv.folds = 10 |
| XGBoost | max_depth=1, eta=0.4,nround=20, objective = "binary:logistic", min_child_weight = 1, subsample = 1, colsample_bytree = 1,gamma = 0.001 |

Supplementary 2. Model parameters

Supplementary 3. VIF value

| Feature | VIF |
| --- | --- |
| Postop-DBIL | 2.029455 |
| Postop-INR | 1.383764 |
| HDL | 1.739663 |
| Postop-ALT | 1.078030 |
| NK | 1.105593 |
| FK506 | 1.060379 |
| Na^+^ | 1.199766 |
| Operation time | 1.084349 |
| Anhepatic phase | 1.063704 |
| Induction regimen | 1.106634 |
| ICU stay | 1.053339 |
